# Breast epidermoid cyst: a case report

**DOI:** 10.3389/fonc.2024.1473227

**Published:** 2024-11-14

**Authors:** Xiaoxiao Dong, Jian Sun, Dong Song, Jinxiu Ma, Xiaozhen Wang

**Affiliations:** Department of Breast Surgery, General Surgery Center, The First Hospital of Jilin University, Changchun, Jilin, China

**Keywords:** breast tumor, epidermoid cyst, diagnosis, differential diagnosis, pathology

## Abstract

An epidermoid cyst is a common benign skin lesion that can occur in any part of the body. However, epidermoid cysts occurring in the breast are extremely rare, rarely reported, and more prone to complications such as infection, rupture, and malignant alteration. Here, we report a case of a 50-year-old woman with a mammary epidermoid cyst. The patient noticed a mass of the left nipple during bathing. A puncture test was performed considering the epidermoid cyst. Moreover, local extended resection of the nipple and areola was performed. Postoperative pathological findings confirmed the diagnosis of a mammary epidermoid cyst. After 2 years of postoperative follow-up, the patient had no surgery-related complications and no signs of tumor recurrence.

## 1 Introduction

Epidermoid cyst, also known as epidermal cyst, interstitial cyst, or funnel cyst, is a common benign skin lesion (1). It is located in the dermis and/or subcutaneous tissue and is one of the most common skin cysts. These cysts are often found on areas of the body with hair.

Epidermoid cysts occurring in the mammary glands are clinically extremely rare and rarely reported. To date, fewer than 40 cases have been reported. The disease is not specific in its clinical manifestations and signs, and it is difficult to distinguish it from other benign and malignant masses of the breast. Therefore, imaging and puncture pathology examination are crucial for the preoperative diagnosis of the disease. Local extended resection containing the mass is the best treatment for breast epidermoid cyst and the primary measure to prevent complications such as infection and malignant alteration (2). Here, we report a case of a mammary epidermoid cyst. Considering the paucity of clinical cases, limited literature reports, and little information based on single experiences, this case report aims to contribute to the knowledge about breast epidermoid cysts.

## 2 Case report

**Figure 1:**
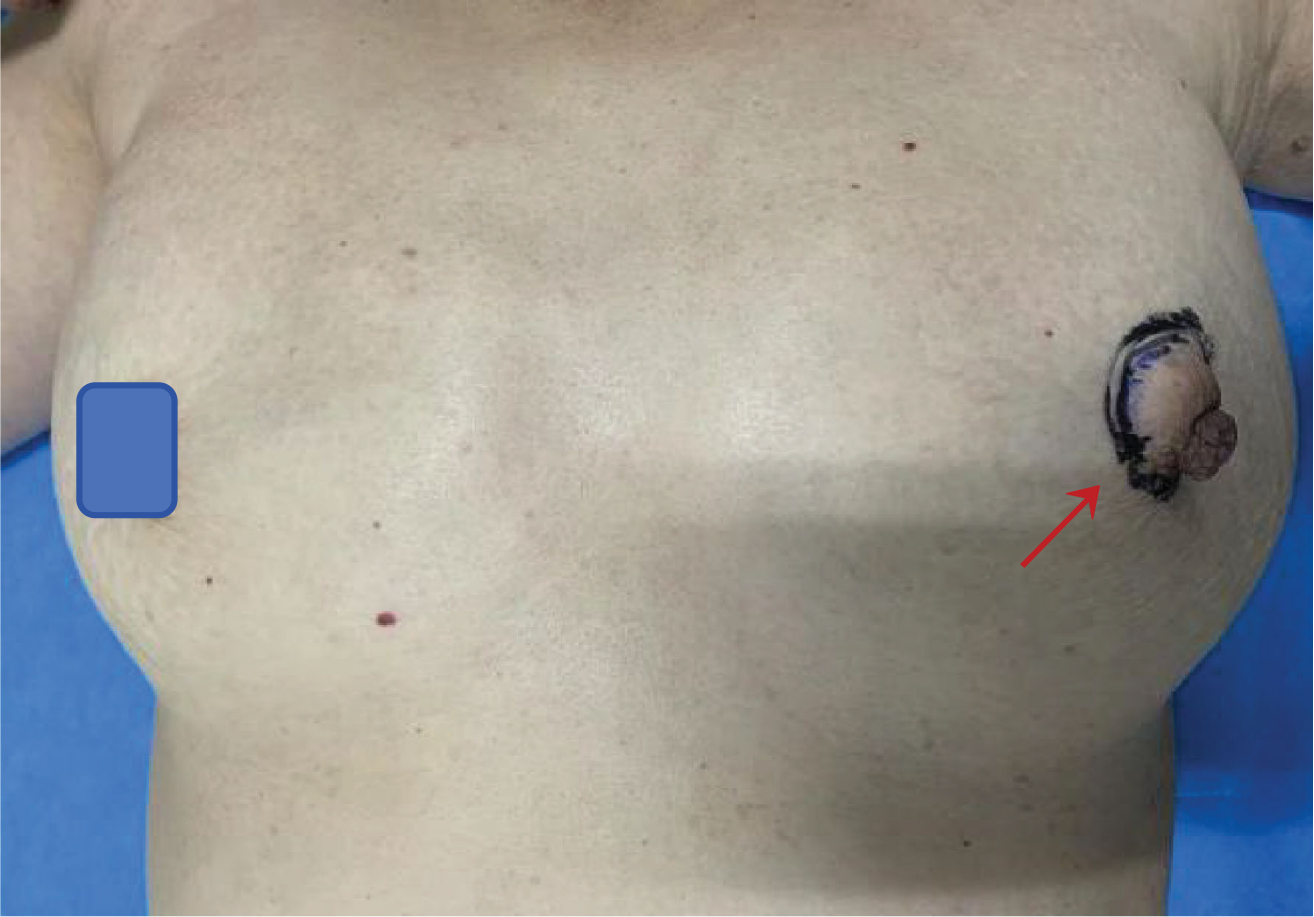
Preoperative anterior view of the patient with a breast epidermoid cyst (red arrow).

**Figure 2:**
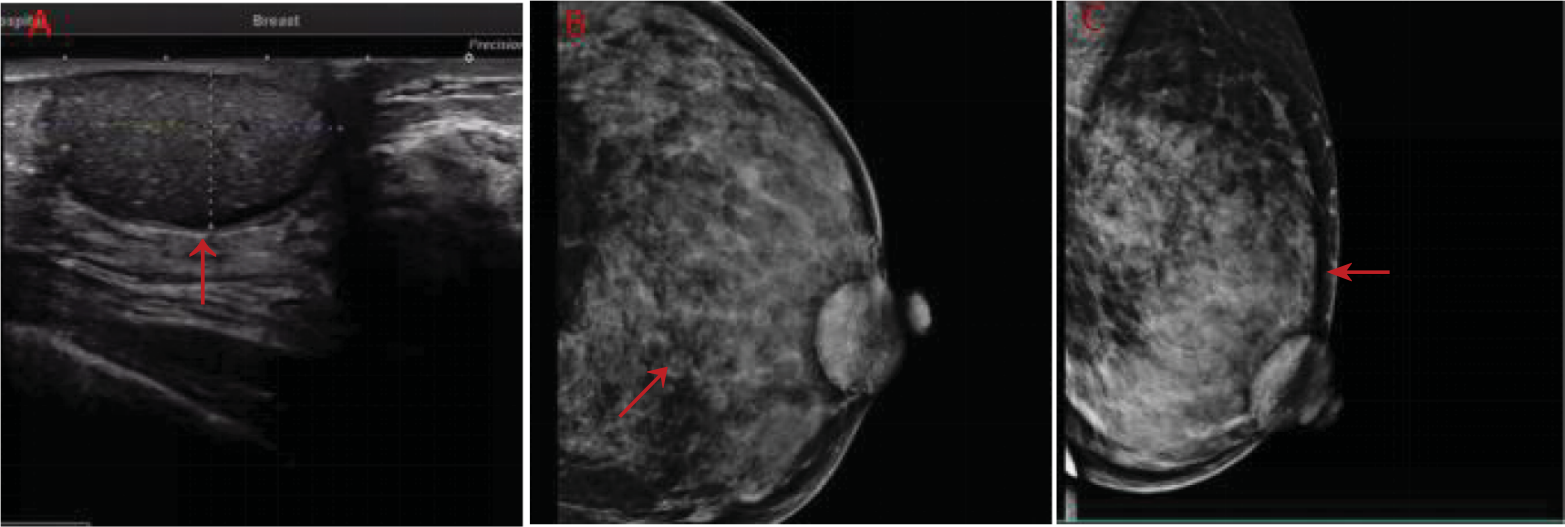
**(A)** Color ultrasonography evaluation of the patient before surgery. **(B, C)** Preoperative images using molybdenum target mammography (red arrows indicate lesions).

**Figure 3:**
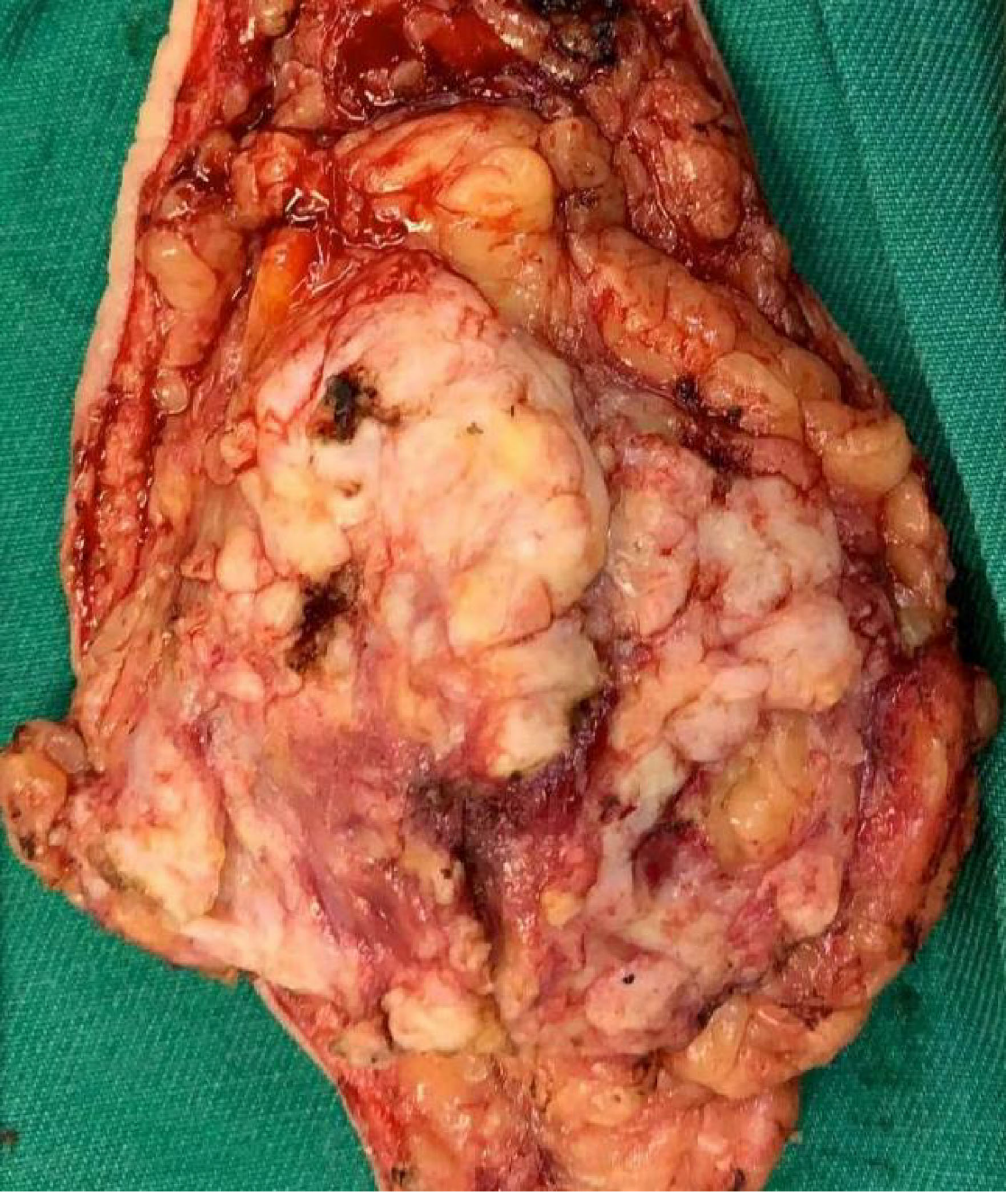
General view of the postoperative specimens.

**Figure 4:**
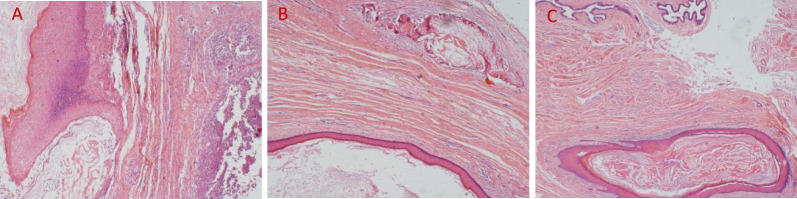
**(A)** Local epithelial breakage, ulcer formation, and microabscess of the interstitium outside the cyst wall (HE, ×40); **(B)** extracystic interstitium showing keratin overflow and foreign body giant cell reactions (HE, ×40); **(C)** the cyst cavity covered with a squamous epithelium and keratinocytes visible in the cyst cavity (HE, ×40).

A 50-year-old woman with a mass near the left nipple was admitted on 3 January 2022. Four months before, the patient accidentally found the mass near the left nipple. The mass had a soft texture and poor mobility. It was approximately 2.0 × 2.0 cm in size and showed no swelling, pain, or ulceration. It was left without any treatment. In the past 2 months, the patient visited our hospital, and the size of the mass had increased to approximately 3.0 × 3.0 cm (**Figure 1**). The patient had no history of surgery and trauma and had a normal menstrual history. There was no remarkable personal or family history. Physical examination showed left–right breast asymmetry, with the left breast slightly greater than the right one. None of the breasts showed skin redness, orange peel signs, nipple depression, or dimpling. The bulge of the mass was visible in the left nipple areola area and was seriously adherent and not mobile. A mass of approximately 3.0 × 3.0 cm was palpable directly behind the left nipple and areola area. It was soft, with a clear boundary and poor mobility on palpation. No significant mass was palpable in the right mammary gland. No enlarged lymph nodes were found in the bilateral axillary and supraclavicular regions. Color Doppler ultrasound examination revealed a low echo mass measuring 3 cm × 1.5 cm on the inner side of the left breast nipple, with a clear boundary and regular morphology, strong echo light spots, and blood flow signal around the periphery. Based on ultrasound, a diagnosis of left breast mass BI-RADS 4 class 4a was made (**Figure 2A**). Molybdenum target mammography revealed an isodense mass with a clear margin in the left areola area. There was no obvious mass and suspicious calcification in the right breast. In both breasts, skin and nipple shadows were normal. Mammography diagnosis was a mass in the left areola area: BI-RADS 4A (**Figures 2B, C**). In the punctured tissue, there were a lot of cuticular samples, a little dermal collagen-like tissue, and a few broken squamous epithelium cells, so an epidermoid cyst was considered. The preoperative diagnosis was a breast epidermoid cyst. Local extended resection of the mass of the nipple was conducted, and the operation went smoothly. However, during the resection, it was found that the internal soybean residue-like substance involved a larger area than what was seen on actual imaging with a foul odor. In order to remove it completely and reduce the risk of recurrence, we had to remove a larger area to ensure the safety of oncology. A general overview of the postoperative specimens is shown in **Figure 3**: a volume measuring 11 cm × 6.5 cm × 2 cm with a skin area of 11 cm × 6.5 cm and a nipple diameter of 1.7 cm; adjacent to the skin, a cystic mass with a volume of 2.5 cm × 2.5 cm × 1.5 cm was seen with a tofu-like residue and a suspicious sinus tract. Microscopic assessment (**Figure 4**) revealed an epidermoid cyst, a local rupture with a foreign body giant cell reaction and a secondary inflammatory reaction, and a local large neutrophil infiltration with the formation of microabscess. The postoperative pathological evaluation confirmed an epidermoid cyst in the left breast. The patient recovered well after surgery and had annual breast color ultrasound and molybdenum target mammography of the surgical site. After 2 years of postoperative follow-up, the patient had no associated complications and no signs of tumor recurrence.

## 3 Discussion

An epidermal cyst is a common, benign tumor of the skin (3), usually located in the dermis or subcutaneous tissue, and is a cystic mass composed of epidermal cells (4). Among epidermal cysts, epidermoid cyst of the breast is an extremely rare benign disease. The clinical manifestations and signs of breast epidermoid cysts are not specific, and they can easily be misdiagnosed as cancer or fibroadenoma. The disease often occurs in women from 20 to 50 years of age, and it is rare in men. The clinical presentation is mostly unilateral, and the cyst usually manifests as a painless mass, but pain may occur in case of secondary infection or rupture. The most common complications of breast epidermoid cysts include spontaneous rupture of the cyst, leading to the release of keratin into the surrounding tissues and causing a granuloma and infection (5).

The risk factors and pathogenesis of breast epidermoid cyst have not been fully clarified but may include some of the following: 1) excessive squamous cell hyperplasia caused by hair follicle occlusion (6); 2) squamous metaplasia of the columnar epithelium caused by fibroadenomas or fibrocystic lesions (7); 3) history of trauma (8) (such as needle biopsy or breast augmentation), leading to implantation of epidermal cells deep into the breast; and 4) abnormal development of primitive embryonic cells remaining in skin attachments (8). In this patient, abnormal development of congenital residual primitive embryonic cells was considered because she had no previous history of trauma, surgery, or breast mass.

The imaging examination encompassed mainly color Doppler ultrasound and mammography. Breast ultrasound and mammography have certain value in the diagnosis of breast epidermoid cyst, but the disease often lacks specific manifestations, so a clear preoperative diagnosis is still difficult (3, 9). Color Doppler ultrasound displays different echoes due to differences in tissue maturity and capsule stratum corneum content, but more round or oval, morphological rules, clear boundaries of low echo solid or mixed mass, part of the interior are echoes of concentric circles or onion skin (9) can be accompanied by tiny calcification, mass without blood flow signal. However, if the epidermoid cyst is accompanied by rupture and infection and a secondary foreign body reaction forms a granuloma or an abscess, the boundary cannot be clearly identified and the blood flow signal can be detected at the place of the granuloma formation. Mammography usually presents a well-circumscribed round mass with uniform and high density, which is indistinguishable from other benign lesions such as solid cysts and adenomas. In addition, relevant literature reports that breast MRI images demonstrated the lamellated nature of the lesions (10), but there are almost no reports on the MRI characteristics of this disease.

Pathology is the gold standard for confirming a breast epidermoid cyst. The main pathological findings of breast epidermoid cysts are generally consistent with those of epidermoid cysts in other sites. Generally, the pathological findings include a round mass that is gray-white or brown-black, with a complete capsule and the capsule wall made of compound squamous epithelium cells; composed of keratin and cholesterol; without hair follicles, sweat glands, and sebaceous glands; and surrounded by a giant cell reaction and an inflammatory reaction. It is difficult to diagnose breast epidermoid cyst before surgery, and this disease needs to be differentiated from breast lesions such as fibroadenoma and breast cyst. 1) Breast fibroadenoma: It grows in the gland with smooth boundaries. It has a uniform internal echo, a light rear echo enhancement effect, and visible blood flow signals on ultrasound. In contrast, there is no blood flow signal inside a breast epidermoid cyst. 2) Cyst of galactostasia: It is mostly formed during lactation or after lactation. It is found in a deep location and presents as a round or oval nodular shadow, with a thin and smooth capsule, good sound transmission, and rear echo enhancement. 3) Breast cancer: It is mostly a painless mass, hard in quality with a smooth surface, which has poor mobility on palpation and poor demarcation from the surrounding tissue. It is located near the nipple–areola area. It may easily develop axillary lymph node metastasis. On ultrasound, morphological irregularities and blood flow signals are visible inside.

Breast epidermoid cyst is a benign disease, and surgical removal of the lesion is the preferred and effective treatment. Breast epidermoid cysts can be treated using surgery with a good prognosis, and there is no need for radiation therapy, chemotherapy, or other adjuvant treatment measures. However, the disease has the risk of recurrence and malignant changes (7, 11). Previous studies have reported that the malignancy rate of epidermoid cysts on the body surface is 0.045%, but the malignancy rate of epidermoid cysts of the breast can be as high as 0.5% (12), which may be related to incomplete intraoperative tumor resection or local rupture of the cyst. Therefore, the surgical method is usually adopted to completely remove the cyst and its cyst wall through incision. This allows for a clear pathological diagnosis and can prevent complications such as infection and malignant alterations (2, 3). Therefore, patients need regular and long-term follow-up. In our patient, a mass was found in the left nipple–areola area, with serious adherence and poor mobility. If the nipple and areola are preserved, complete removal of the cyst and its cystic wall cannot be achieved. The operation also confirmed our preoperative prediction. Therefore, there was clear communication with the patient before surgery regarding the removal of the nipple and areola. The patient agreed to the nipple–areola resection, so we implemented local resection of the nipple–areola. The following surgical points should be considered: it is important not to rupture the cyst wall during the operation; the cyst and its wall must be completely removed; and expanded resection should be performed if necessary to reduce the risk of recurrence. During the 2-year follow-up, the patient did not show signs of local recurrence and distant metastasis.

## 4 Conclusion

Given the malignant potential of breast epidermoid cysts, close postoperative follow-up is necessary. Future studies should focus on reporting more cases and clinical data to better understand the behavior, prognosis, and optimal management strategies for breast epidermoid cysts.

## Data Availability

The raw data supporting the conclusions of this article will be made available by the authors, without undue reservation.
